# Supplementary material for: Disentangling genetic effects on transcriptional and post-transcriptional gene regulation through integrating exon and intron expression QTLs
Source: Nat Commun. 2024 May 6;15:3786. doi: 10.1038/s41467-024-48244-x (PMC11074126; doi:10.1038/s41467-024-48244-x)
Supplement: Supplementary file 1 — Supplementary Information [file 41467_2024_48244_MOESM1_ESM.pdf]

Disentangling genetic effects on transcriptional and post-transcriptional gene regulation through integrating exon and intron expression QTLs

Anneke Brümmer and Sven Bergmann

Supplementary Figures

Supplementary Figure 1: Gene quantification based on exonic and intronic RNA-Seq reads.

Supplementary Figure 2: Replication of top cis-exQTLs, cis-inQTLs and cis-ex-inQTLs in the CoLaus and Geuvadis data sets.

Supplementary Figure 3: Sharing between top cis-QTLs and between conditional cis-QTL signals.

Supplementary Figure 4: Trans-QTL associations for cis-QTLs of transcription factors, RNA-binding proteins, and miRNAs.

Supplementary Figure 5: Colocalization between GWAS variants and top cis-QTLs based on linkage disequilibrium (LD).

Supplementary Figure 6: Colocalization between top cis-QTLs and GWAS variants for 78 traits with at least 25 colocalizations.

Supplementary Figure 7: Additional examples for cis-QTLs from different clusters colocalizing with GWAS variants.

Supplementary Figure 8: Cis-QTLs for exon and intron expression levels and their ratio detected in 78 fibroblast samples from Delaneau et al. (2019).

Supplementary Notes

Supplementary Note 1: Comparison of gene expression levels based on exonic and intronic RNA-Seq reads.

Supplementary Note 2: Replication of top cis-QTL signals in CoLaus and Geuvadis data sets.

Supplementary References

Supplementary Figure 1

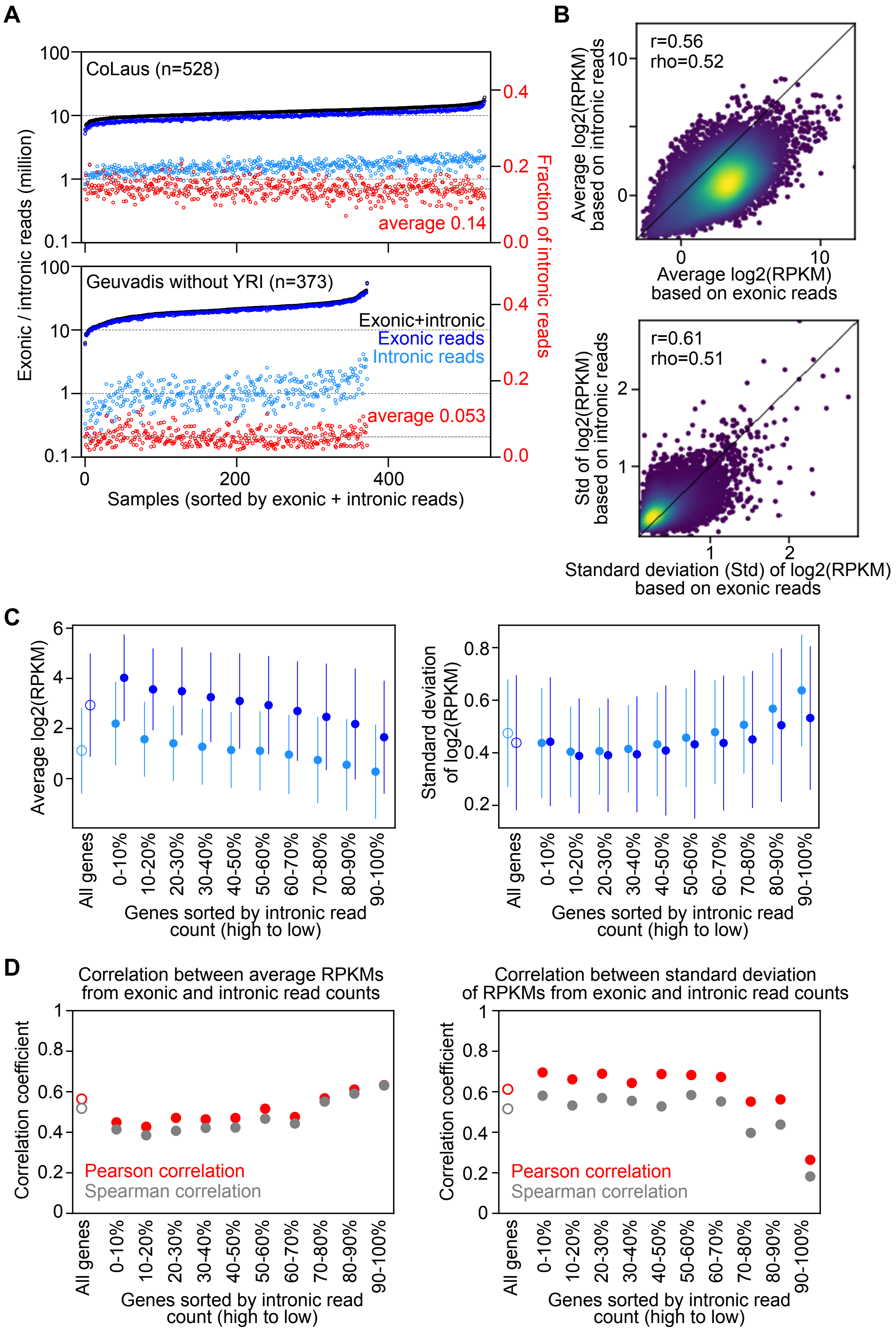

Supplementary Figure 1: Gene quantification based on exonic and intronic RNA-Seq reads. (A) Number of RNA-Seq reads mapping uniquely to exons (blue), introns (light blue), or both (black) for samples in the Colaus data set (top panel) and the Geuvadis, without individuals from Yoruba (YRI), Africa, data set (bottom panel). Samples are sorted by number of reads mapping to exons and introns. The second y-axis (right) indicates the fraction of reads mapping uniquely to introns (red) with the average over all samples indicated. (B) Comparison of averages (top panel) and standard deviations (bottom panel) of gene expression levels quantified as log2 RPKM (read per kilobase per million mapped reads) from exonic (x-axes) or intronic (y-axes) uniquely mapped RNA-Seq reads. Averages and standard deviation were calculated across samples for each gene (9020 genes in total). Pearson (r) and Spearman (rho) correlation coefficients are indicated. (C) Comparison of average (left panel) and standard deviation (right panel) of average log2 RPKM levels of genes quantified from exonic (blue) or intronic (lightblue) reads. Mean (circle) and standard deviation (error bar) are indicated for all genes (first circle of each colour) and for 10 groups of genes with decreasing number of intronic reads. Each group comprises 900 genes. (D) Pearson (red) and Spearman (grey) correlation coefficients between averages (left panel) and standard deviations (right panel) of exonic and intronic log2 RPKM levels per gene, for all genes (first circle of each colour) and for the 10 groups of genes with decreasing numbers of intronic reads from (C).

Supplementary Figure 2

A

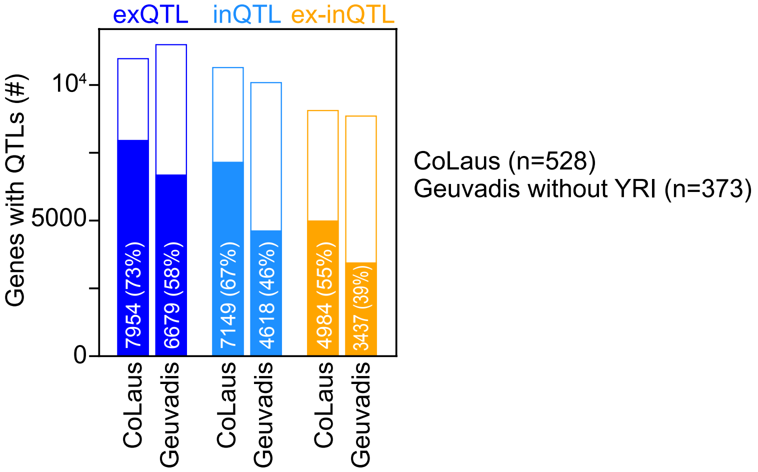

B

1. Replication of CoLaus QTLs in Geuvadis data set

		Tested in both	Significant in CoLaus	%	Significant in both	%	Same top QTL in both	Shared top QTL signal	%
All genes	exQTLs	10847	7876	73%	5952	76%	946	2955	66%
	inQTLs	9887	6698	68%	4232	63%	574	2107	63%
	ex-inQTLs	8619	4831	56%	2979	61%	377	1558	65%
Genes with high read count	exQTLs	5423	4143	76%	3105	74%	467	1569	66%
	inQTLs	4943	3825	77%	2442	64%	315	1185	61%
	ex-inQTLs	4309	2789	65%	1726	62%	198	888	63%
Genes with low read count	exQTLs	5423	3727	69%	2843	76%	479	1383	65%
	inQTLs	4943	2870	58%	1789	62%	259	921	66%
	ex-inQTLs	4309	2040	47%	1242	61%	178	669	68%

2. Replication of Geuvadis QTLs in CoLaus data set

		Tested in both	Significant in Geuvadis	%	Significant in both	%	Same top QTL in both	Shared top QTL signal	%
All genes	exQTLs	10847	6679	62%	5952	93%	946	2955	66%
	inQTLs	9887	4618	47%	4232	93%	574	2107	63%
	ex-inQTLs	8619	3437	40%	2979	88%	377	1558	65%
Genes with high read count	exQTLs	5423	3306	65%	3105	94%	467	1569	66%
	inQTLs	4943	2584	61%	2442	95%	315	1185	61%
	ex-inQTLs	4309	1914	62%	1726	90%	198	888	63%
Genes with low read count	exQTLs	5423	3081	57%	2843	92%	479	1383	65%
	inQTLs	4943	1970	40%	1789	91%	259	921	66%
	ex-inQTLs	4309	1462	34%	1242	85%	178	669	68%

C

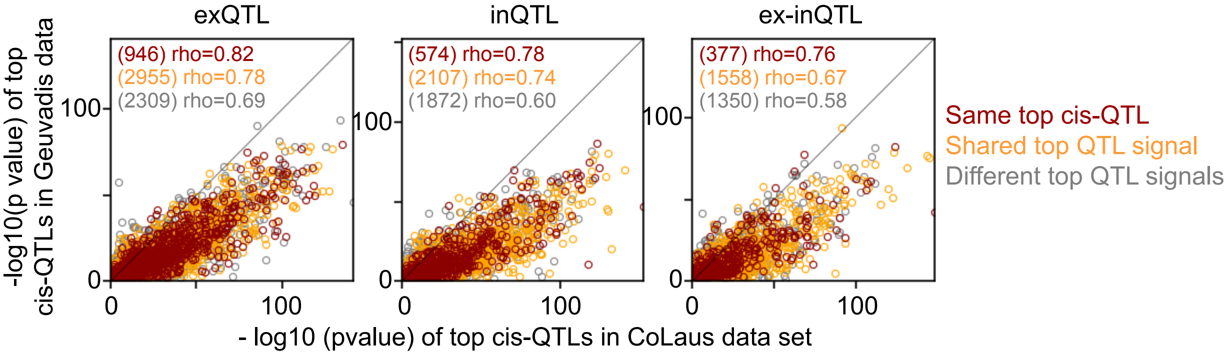

Supplementary Figure 2: Replication of top cis-exQTLs, cis-inQTLs and cis-ex-inQTLs in the CoLaus and Geuvadis data sets.

(A) Number and percentage of genes with cis-QTLs (filled bars) of tested genes (full bars) for exQTLs (blue), inQTLs (light blue) and ex-inQTLs (orange) for the CoLaus data set (n=528) and for Geuvadis data set, excluding samples from African individuals (n=373). (B) Replication of cis-QTLs: 1. detected in the Colaus data set and replicated in the Geuvadis data set and 2. detected in the Geuvadis data set and replicated in the Colaus data set, for all genes (top panel for each replication), genes with high RNA-Seq read counts (middle panels) and genes with low read counts (bottom panels). Percentages are calculated relative to the previous columns, except for the last column, which indicates the percentage of genes with same and shared cis-QTLs relative to genes with significant QTLs in both data sets. Percentages in blue and green are very different between replications 1. and 2., and percentages in red are very different between genes with high and low read counts for each replication (1. and 2.) (C) Scatter plots of -log10(p-values) in the Colaus (x-axis) and Geuvadis (y-axis) data sets for cis-exQTLs (first panel), cis-inQTLs (second panel), cis-ex-inQTLs (last panel). The numbers and Spearman correlation coefficients for top cis-QTLs with same positions (dark red), shared signals (orange), and different, not-shared positions (grey) are indicated.

Supplementary Figure 3

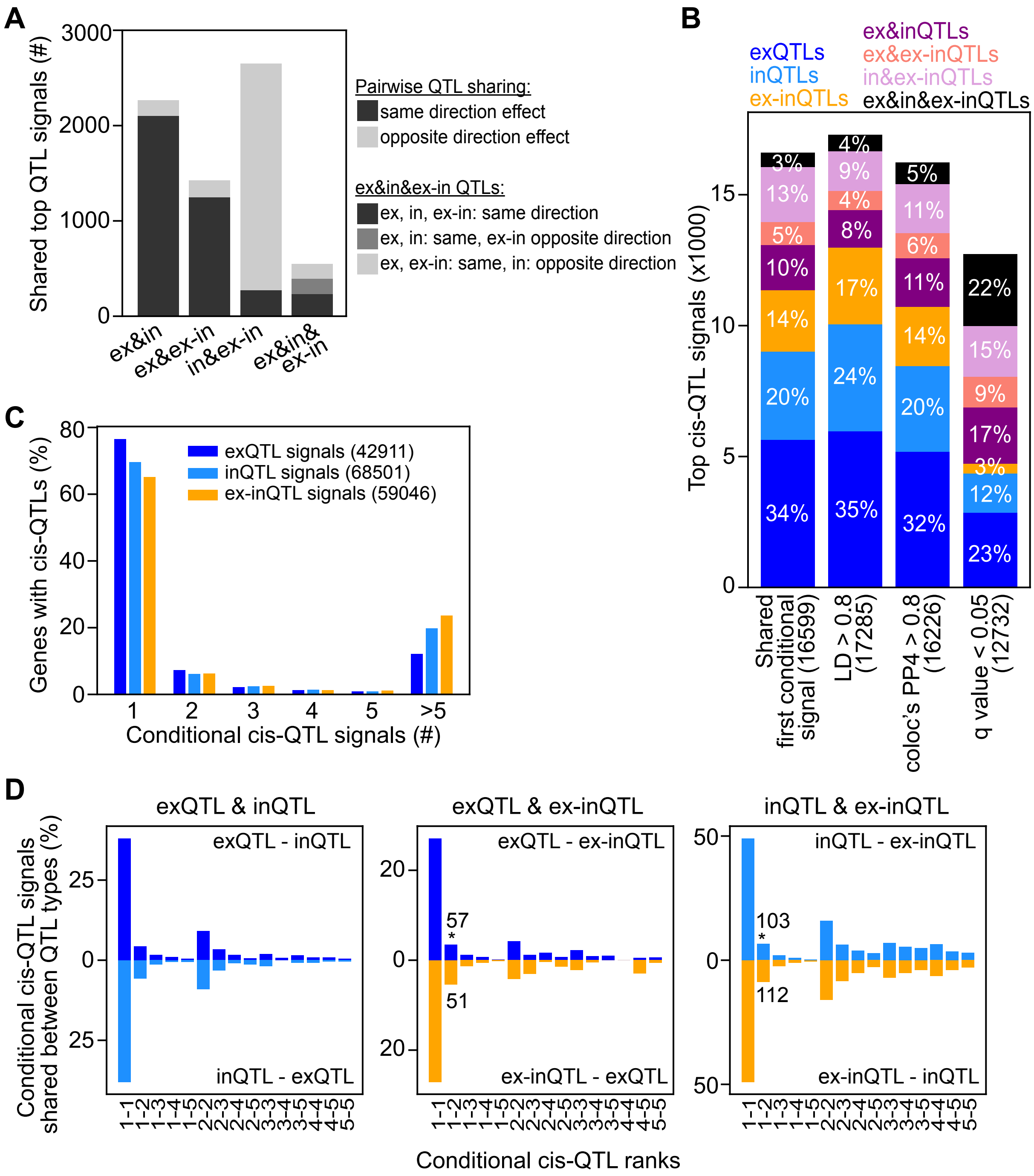

Supplementary Figure 3: Sharing between top cis-QTLs and between conditional cis-QTL signals. (A) Number of genes with shared top cis-QTL signals with effects in the same or opposite direction. In case of sharing between all three QTL types, the numbers are further divided into all effects having the same direction, exQTL and inQTL effects having the same direction and the ex-inQTL effect the opposite direction, or exQTL and ex-inQTL effects having the same and the inQTL effect the opposite direction. (B) Number of distinct top cis-QTL signals obtained using different methods for defining sharing between cis-QTLs: first bar: sharing based on first conditional cis-QTL signals (used in this study; see Methods), second bar: sharing based on linkage disequilibrium (LD) requiring $r^2 > 0.8$, third bar: sharing based on posterior probability for colocalized association (PP4) >0.8 calculated using coloc [1], fourth bar: sharing based on q value [2] < 0.05 for nominal p values of top cis-QTLs for both directions of sharing between QTL types. For each stacked bar, the

percentages of cis-QTL signals shared between different cis-QTL types (colour coded) are indicated, and the total number of independent cis-QTL signals is indicated in parenthesis on the x-axis. (C) Percentage of genes with certain numbers of conditional cis-QTL signals (see Methods). The total number of conditional cis-QTL signals of each type is indicated in parenthesis. (D) Percentage of conditional cis-QTL signals of ranks 1 to 5 (indicated on the x-axis) that are shared between QTL types, as indicated on top of each panel. Bars in top direction show the comparison between ranks of two QTL types as indicated on top right, while bars in bottom direction show the comparison between the same QTL types, but with reversed ranks, indicated at bottom right. Numbers on the x-axis indicate the rank of the first and second QTL type, which are indicated at top right and bottom right of each panel. The number of shared conditional QTL signals is indicated in case of significantly different proportions between cis-QTLs with original and reversed ranks. *, $p<0.05$, two-sided Fisher's exact test ($p=0.025$ and $p=0.04$ for sharing of conditional cis-ex-inQTLs with cis-exQTLs and with cis-inQTLs, respectively).

Supplementary Figure 4

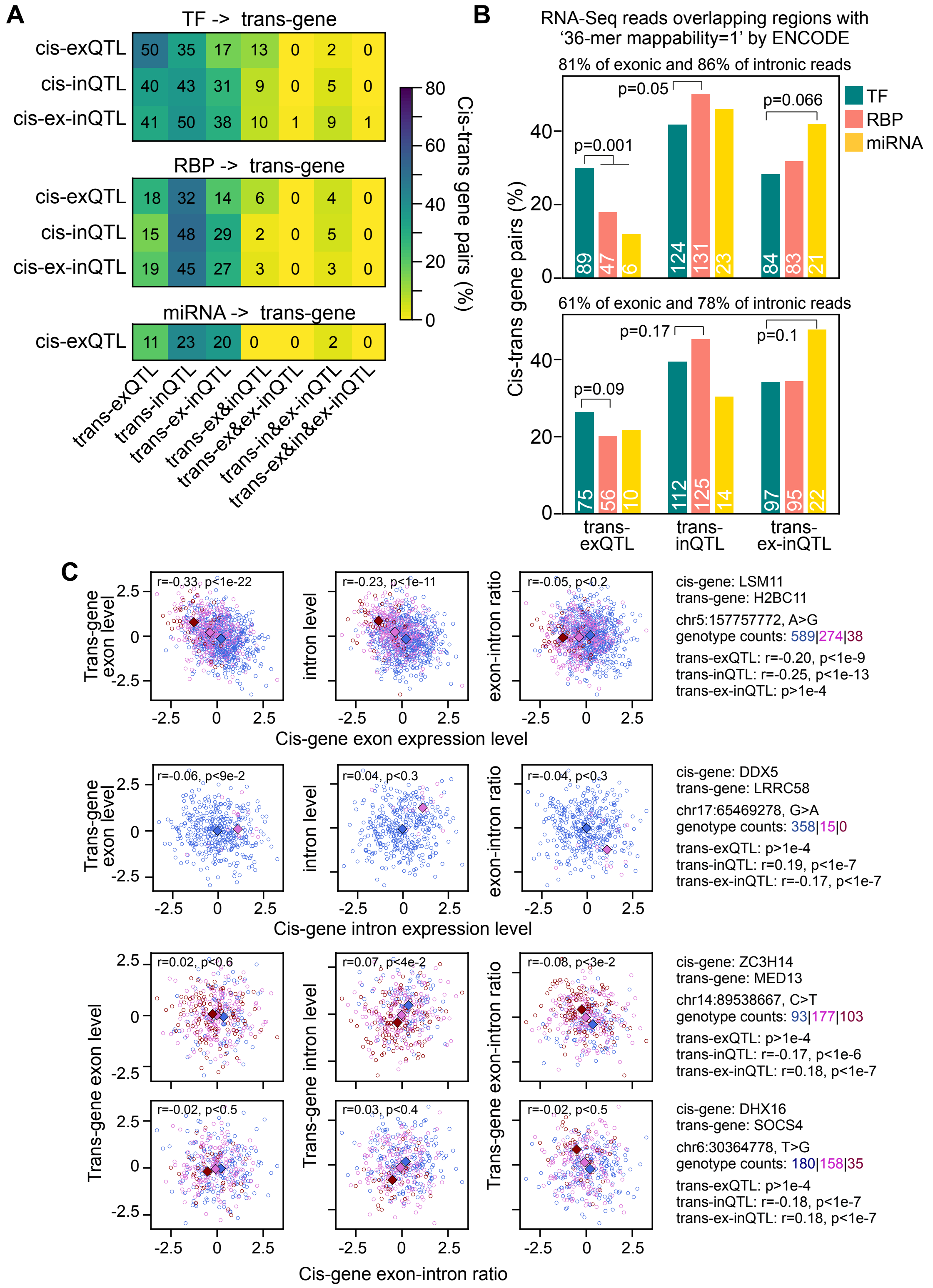

Supplementary Figure 4: Trans-QTL associations for cis-QTLs of transcription factors, RNA-binding proteins, and miRNAs.
(A) Heatmap of percentages of cis-trans-QTL gene pairs for cis-exQTLs, cis-inQTLs and cis-ex-inQTLs of transcription factors (TFs; top panel) and RNA-binding proteins (RBPs; middle panel) and miRNAs (bottom panel) separated by type of trans-association (indicated on the y-axis). The number of cis-trans-QTL gene

pairs of each type are indicated. (B) Percentages of detected trans-QTL associations of different types for transcription factors (TF), RNA-binding proteins (RBP), and miRNAs, when considering only RNA-Seq read pairs for which at least one read aligned with half of its length (upper panel) or with its full length (lower panel) to a genomic region that was annotated with 36-mer mappability =1 by ENCODE (see Methods). The number of RNA-Seq read pairs that remained after discarding reads that did not fulfil the above requirements is indicated above each panel. The numbers of trans-QTL associations are indicated at the bottom of each bar. P values are calculated using two-sided Fisher's exact test. (C) Examples for cis-regulated RBPs (one per row) associated with another gene in trans through a cis-exQTL (top row), a cis-inQTL (middle row) or cis-ex-inQTLs (bottom two rows). Shown are scatter plots of the RBP's normalised expression values (x-axis) and the normalised exon levels (left panels), intron levels (middle panels) and their ratios (right panels) for the trans-associated gene (y-axis). Information on the involved genes, QTL variant, and correlation coefficients and nominal p values of trans-QTL associations are indicated on the right. Pearson correlation coefficients with p values between the expression levels/ratios of the RBPs and their trans-associated genes are indicated inside each panel. Circles of different colour represent individuals with different genotypes, and coloured squares indicate the median values for individuals with each genotype.

Supplementary Figure 5

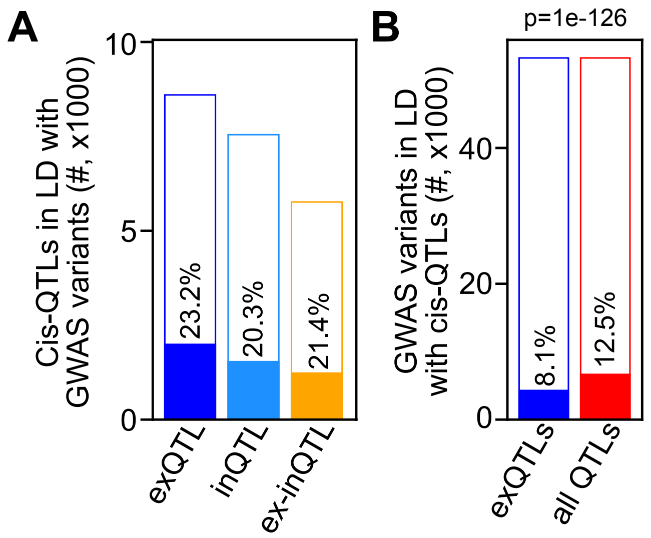

Supplementary Figure 5: Colocalization between GWAS variants and top cis-QTLs based on linkage disequilibrium (LD).
(A) Number and percentage of top cis-QTLs in strong LD ($r^2 > 0.8$) with GWAS variants (filled bars) of tested QTLs (full bars) for different QTL types. (B) Number and percentage of GWAS variants in strong LD ($r^2 > 0.8$) with top cis-QTLs (filled bars) of tested GWAS variants (full bars) for exQTLs (blue bar) and all QTL types (red bar). P value was calculated using two-sided Fisher's exact test.

Supplementary Figure 6

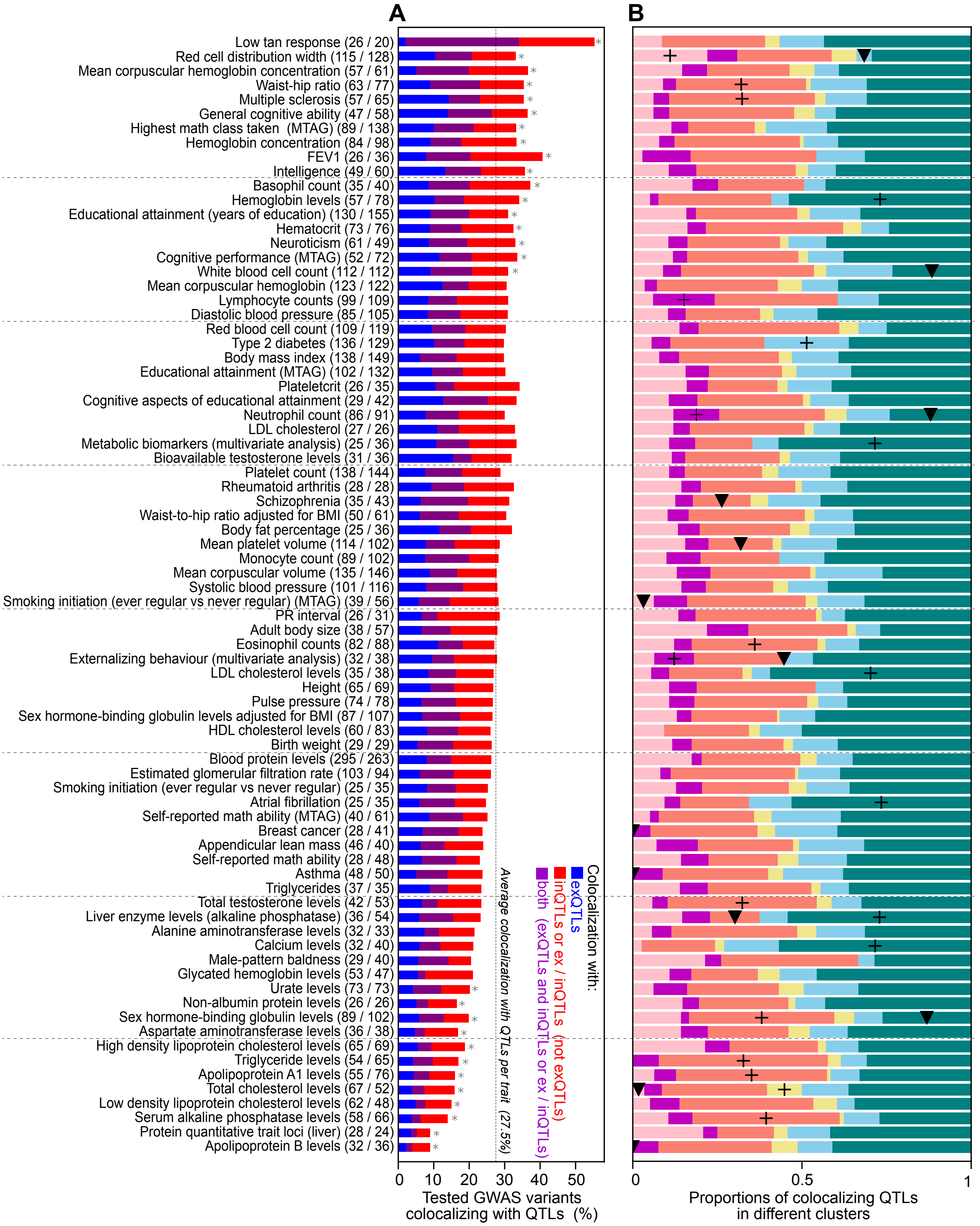

Supplementary Figure 6: Colocalization between top cis-QTLs and GWAS variants for 78 traits with at least 25 colocalizations.

(A) Proportion of tested GWAS variants colocalizing with top cis-QTLs for 78 GWAS traits (indicated at right) with at least 25 colocalizations. GWAS traits are sorted by the significance of the colocalization with cis-QTLs

(calculated using two-sided Fisher's exact test compared to all 78 GWAS traits, *: $p<0.05$). The number of colocalizing GWAS variants and the number of colocalizing cis-QTLs are indicated in parenthesis. The percentage of GWAS variants colocalizing with only exQTLs, or not with exQTLs, or with exQTLs and another QTL type are indicated as stacked blue, red, and purple bars, respectively. (B) Proportion of colocalizing top cis-QTLs with quantified effects on all three gene expression measures that are assigned to certain clusters (colour coded as shown in Figure 4D) according to their relative effect sizes. A plus or triangle symbol indicates an enriched or depleted colocalization, respectively, with cis-QTLs of a certain cluster compared to cis-QTLs assigned to that cluster and colocalizing with any trait ($p<0.05$, calculated using two-sided Fisher's exact test).

Supplementary Figure 7

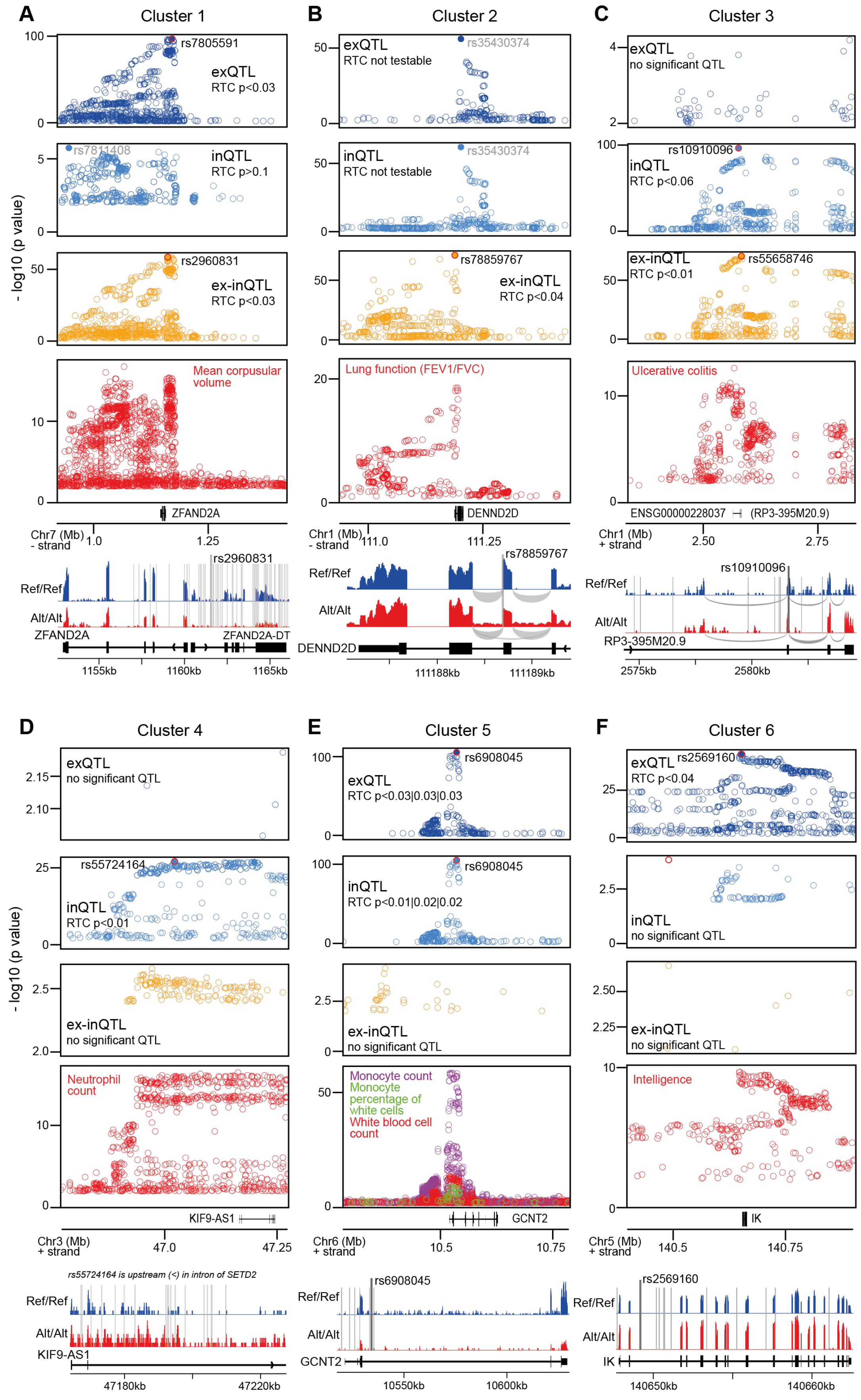

Supplementary Figure 7: Additional examples for cis-QTLs from different clusters colocalizing with GWAS variants.

In each subfigure, the top three panels show, in a region of 500kb around the top-cis-QTL (x-axis), the -log10 nominal p-values (<0.01) for cis-QTL associations with exon levels (dark blue), intron levels (blue) and exon-intron-ratios (orange), and the fourth panel shows the -log10 p-values for the GWAS trait associations (red). The rsID of the top cis-QTL(s) are indicated (in black, if colocalized with the GWAS trait variants, or in grey if not). Colocalization via the RTC method (see Methods) is only testable for QTLs and GWAS variants within the same region surrounded by recombination hotspots. The bottom panel shows examples for RNA-Seq read distributions at the associated gene (or a region thereof) from two homozygous individuals, one with reference (Ref/Ref; blue) and one with alternative (Alt/Alt; red) genotype, for the cis-QTL variant. The positions of the top cis-QTL as well as cis-QTLs sharing the top QTL signal are indicated with thick or thin lines, respectively.

(A) A cis-QTL from cluster 1, associated with *ZFAND2A* and colocalizing with a GWAS variant for mean corpuscular volume. The top cis-QTLs for exon levels and exon-intron-ratios are shared, while the cis-QTL signal for intron levels is different and does not colocalize with the GWAS trait variants. The top cis-ex-inQTL is located in the first intron of the upstream non-coding gene, ZFAND2A-DT, which is annotated as a divergent transcript of ZFAND2A. Individuals homozygous for the alternative cis-ex-inQTL genotype show a reduction in expression of both genes. It cannot be excluded that these genes, that appear to be co-transcriptionally regulated with histone acetylation marks around their gene starts, have additional RNA-interactions after transcription.

(B) A cis-QTL from cluster 2, associated with the exon-intron-ratio of *DENND2D*, a guanyl-nucleotide exchange factor promoting the exchange of GDP to GTP, and colocalizes with GWAS variants for the lung function trait FEV1/FVC. The expression of this gene is also affected by a shared QTL affecting exon and intron levels, which does not colocalize with variants for this GWAS trait. The top cis-ex-inQTL is at the 5' end of the second last intron, between two coding exons, and appears to slow down splicing of the upstream exon, with a probability for skipping that entire coding exon, which is almost 90 nucleotides long.

(C) A cis-QTL from cluster 3, associated with the long, intergenic non-coding RNA (lincRNA) *RP3-395M20.9* and colocalizing with a GWAS variant for ulcerative colitis. The gene has been connected to autoimmune diseases before [3]. The top cis-inQTL is in the second last intron of the lincRNA and appears to increase the rate of splicing of that intron (bottom panel).

(D) A cis-QTL from cluster 4, associated with *KIF9-AS1* and colocalized with a GWAS variant for neutrophil count. There are no significant cis-QTLs for exon levels and exon-intron-ratios. The colocalizing top cis-QTL for intron levels is in the intron of an upstream gene, SETD2, and likely increases transcription slightly, without an increase in the splicing rate, such that it is only detectable at intron levels.

(E) A cis-QTL from cluster 5, associated with *GCNT2* and colocalizing with GWAS variants for three related traits, monocyte count, monocyte percentage of white cells, and white blood cell count. The top cis-QTL is identical for exon and intron levels. It is located in the third (and longest) intron and likely reduces transcription of that gene, detectable at exon and intron levels.

(F) A cis-QTL from cluster 6, associated with *IK* and colocalizing with a GWAS variant for intelligence. The top cis-exQTL is in the second intron of the gene, and likely increases the transcription of that gene.

Supplementary Figure 8

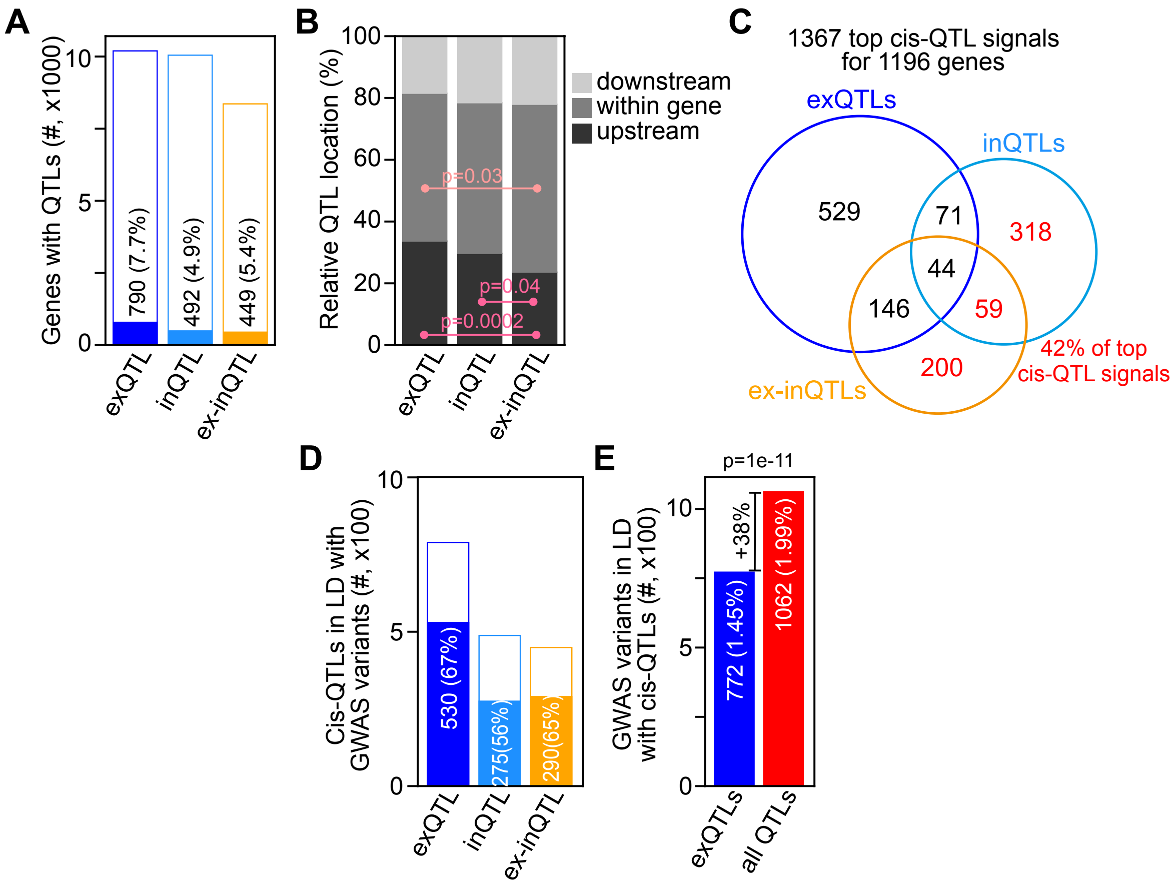

Supplementary Figure 8: Cis-QTLs for exon and intron expression levels and their ratio detected in 78 fibroblast samples from Delaneau et al. (2019).

(A) Number and percentage of genes with cis-QTLs (filled bars) of tested genes (full bars) for different QTL types. (B) Location of top cis-QTLs relative to their associated genes for different QTL types. P values comparing the fractions of genes with different cis-QTLs upstream or within the associated genes were calculated using two-sided Fisher's exact test. (C) Venn diagram showing the sharing between different types of top cis-QTLs, defined based on strong linkage disequilibrium (LD, $r^2 > 0.8$). In total, 1367 distinct top cis-QTL signals were detected for 1196 genes. The percentage of top cis-QTL signals not shared with top cis-exQTLs is indicated in red. (D) Number and percentage of top cis-QTLs in strong LD ($r^2 > 0.8$) with GWAS variants (filled bars) of tested QTLs (full bars) for different QTL types. (E) Number and percentage of GWAS variants in strong LD ($r^2 > 0.8$) with top cis-QTLs (filled bars) of tested GWAS variants for exQTLs (blue bar) and all cis-QTL types (red bar). P value is calculated using two-sided Fisher's exact test. The percentage increase in GWAS variants in strong LD with cis-QTLs when considering all cis-QTL types as opposed to only exQTLs is indicated.

Supplementary Note 1

Comparison of gene expression levels based on exonic and intronic RNA-Seq reads

The number of intronic reads is around 1-2 million reads on average for samples of the CoLaus and Geuvadis data sets (Supplementary Figure 1A), which is about 10 times lower than the number of exonic reads (on average ~10 million in the CoLaus data set and ~15-20 million in the Geuvadis data set). To verify if the calculated gene expression levels based on intronic reads are biologically meaningful, we compared them with those based on exonic reads for each gene. As intronic reads are depleted in polyA-selected RNA-Seq data, the average (across samples) gene expression levels (quantified as reads per kilobase per million reads, RPKM) from intronic reads are generally lower than gene expression levels from exonic reads (2.2 and 7.6 RPKM based on intronic and exonic reads, respectively). However, we find a good correlation between the average (across samples) exonic and intronic RPKMs for 9020 quantifiable genes (Pearson correlation coefficient 0.56, p=0; Supplementary Figure 1B, top panel), and also between the standard deviations (across samples) of RPKMs for each genes (Pearson correlation coefficient 0.61, p=0; Supplementary Figure 1B, bottom panel). The average standard deviations were similar for exonic and intronic RPKMs (1.36 and 1.39, respectively), indicating larger relative standard deviations for intronic RPKMs (0.64) than for exonic RPKMs (0.18), likely due to the lower number of intronic RNA-Seq reads resulting in less accurate gene expression quantification.

To further evaluate the reliability of RPKM quantifications for low read counts, we separated the genes into 10 groups with decreasing number of intronic reads. We observed decreasing average exonic and intronic RPKM levels for genes with lower intronic read counts (Supplementary Figure 1C, left panel), and increasing standard deviations of exonic and intronic RPKM levels for these genes (Supplementary Figure 1C, right panel), indicating a lower accuracy in the average RPKM levels for genes with low read counts. Nevertheless, the Pearson correlation coefficient between average exonic and intronic RPKMs was >0.42 for all groups of genes (Supplementary Figure 1D, left panel), and >0.5 between the standard deviations of exonic and intronic RPKMs for all except the last group with lowest read counts, where the correlation between standard deviations was only 0.26 (Supplementary Figure 1D, right panel).

Thus, the number of RNA-Seq reads does impact the accuracy of the gene expression quantification, but as exonic and intronic gene expression levels are well correlated, intronic quantifications appear to be biologically meaningful as well in our data set, despite being based on a much lower number of RNA-Seq reads.

Supplementary Note 2

Replication of top cis-QTL signals in CoLaus and Geuvadis data sets

To evaluate the reproducibility of cis-QTL associations for exon and intron levels and for their ratio, we mapped cis-QTLs in two independent data sets, both consisting of RNA-Seq data from lymphoblastoid cell lines (LCLs) and genotypes from European individuals. In particular, we analysed 528 samples of the CoLaus data set [4] and 373 samples of the Geuvadis data set [5], excluding samples from African individuals from Yoruba (YRI). We restricted our analysis to 6.8 million single-nucleotide variants that were genotyped in both data sets. We separately counted RNA-Seq reads mapping uniquely to either exons or introns, and quantified exonic and intronic expression levels for each gene, as well as the ratio of these (see Methods for details). Genes were tested for cis-QTL associations if the median number of uniquely mapping RNA-Seq reads was at least 10 across individuals in a data set. We detected significant (FDR < 5%) exQTLs, inQTLs and ex-inQTLs in cis for 73%, 67% and 55% of testable genes, respectively, in the Colaus data set, and for 58%, 46% and 39% of testable genes, respectively, in the Geuvadis (without YRI) data set (Supplementary Figure 2A).
Overall, the percentage of genes with cis-QTLs was lower for the Geuvadis (without YRI) data set than for the CoLaus data set, likely due to the smaller sample size (71% of CoLaus data set). For both data sets, the percentage of genes with inQTLs was lower compared to the percentage of genes with exQTLs, likely because of the lower number of intronic RNA-Seq reads compared to exonic RNA-Seq reads, and thus a lower accuracy in the measured expression levels of introns compared to exons (see Supplementary Note 1 and Supplementary Figure 1). The percentage of genes with inQTLs and ex-inQTLs was particularly lower in the Geuvadis (without YRI) data set, potentially due to a lower proportion of RNA-Seq reads mapping to introns (on average 0.14 for CoLaus versus 0.053 for Geuvadis data set; Supplementary Figure 1A).

Of the genes that were tested in both data sets and that had significant cis-QTL associations in the CoLaus data set, 76%, 63% and 61% also had significant exQTLs, inQTLs, and ex-inQTLs, respectively, in the Geuvadis data set (Supplementary Figure 2B, first panel, green numbers), while 93%, 93% and 88% of the significantly associated genes in the Geuvadis data set also had significant exQTLs, inQTLs, and ex-inQTLs, respectively, in the CoLaus data set (Supplementary Figure 2B, second panel, green numbers). This indicates that the replication rate of genes with cis-QTLs is considerably larger, when replicating in a larger data set, which allows for detection of more cis-QTL associations compared to a smaller data set (compare blue numbers in top and bottom panels of Supplementary Figure 2B).
The replication rate of genes with cis-QTLs was slightly lower for inQTLs (for CoLaus inQTLs in the Geuvadis data set) and for ex-inQTLs (in both directions of replication). In contrast, of the genes with cis-exQTLs, cis-inQTLs and cis-ex-inQTLs in both data sets, a similar fraction, 66%, 63% and 65%, respectively, had identical top cis-QTL positions or shared top cis-QTL signals in both data sets.

To further understand the effect of the RNA-Seq read count on the detection and replication of cis-QTLs, we separated the genes tested in the CoLaus and Geuvadis data sets into two groups based to their exonic and intronic read counts (specifically the average of their ranks in exonic and intronic read counts). While the detection rate of cis-QTLs was larger for genes with higher read counts compared to genes with lower read count in both data sets (CoLaus: 76% vs. 69% for exQTLs, 77% vs. 58% for inQTLs, 65% vs. 47% for ex-inQTLs, and Geuvadis: 65% vs. 57% for exQTLs, 61% vs. 40% for inQTLs, 62% vs. 34% for ex-inQTLs; compare red numbers in Supplementary Figure 2B), the read count had no influence on the replication rate of genes with cis-QTLs (CoLaus replicated with Geuvadis: 74% vs. 76% for exQTLs, 64% vs. 62% for inQTLs, 62% vs. 61% for ex-inQTLs, and Geuvadis replicated with CoLaus: 94% vs. 92% for exQTLs, 95% vs. 91% for inQTLs, 90% vs. 85% for ex-inQTLs; column right of “Significant in both” in

Supplementary Figure 2B) and on the fraction of cis-associated genes with identical or shared cis-QTL between data sets (61-66% for different QTL types for genes with high read counts and 65-68% for genes with low read counts; last column in Supplementary Figure S2B).
Thus, the sample size and the gene read count both influence the detection rate of cis-QTLs, which then has consequences on the replication rates. Independently of this, the gene read count does not impact the replication rate, indicating the reliability of detected cis-QTLs even for genes with low read counts.

We also used the CoLaus and Geuvadis data sets to estimate the false positive rate (FPR) or type I error of different QTL types assuming that cis-QTL associations detected with the larger data set (CoLaus) are true. In this way, in the Geuvadis data set the FPRs (= FP / [FP + TN], with FP the number of false positive associations and TN the number of true negative associations) were 18.2% for exQTLs, 14.7% for inQTLs, and 15.3% for ex-inQTLs. Thus, the FPRs are similar, and in particular not larger for ex-inQTLs and inQTLs than for exQTLs, indicating the reliability of ex-inQTL and inQTLs proposed in this study. (Notably, the absolute FPRs depend on the specific sample sizes of two data sets.)

The concordance of cis-QTL effects in the CoLaus and Geuvadis data sets, for identical, shared or unrelated cis-QTLs, is also reflected in the strength of correlation of their p values, which is strongest for cis-QTLs with identical positions and weakest for unrelated cis-QTLs for the same gene (Supplementary Figure 2C).

In summary, despite differences in sample size and in the fraction of intronic reads, all types of QTLs appear reproducible across the two independent CoLaus and Geuvadis (without YRI) data sets.

Supplementary References

1. Giambartolomei, C. et al. Bayesian test for colocalisation between pairs of genetic association studies using summary statistics. PLoS Genet. 10, e1004383 (2014).
2. Storey, J. D. The False Discovery Rate: A Bayesian Interpretation and the Q-Value. (2001).
3. Hrdlickova, B. et al. Expression profiles of long non-coding RNAs located in autoimmune disease-associated regions reveal immune cell-type specificity. Genome Med 6, 88 (2014).
4. Sönmez Flitman, R. et al. Untargeted Metabolome- and Transcriptome-Wide Association Study Suggests Causal Genes Modulating Metabolite Concentrations in Urine. J. Proteome Res. 20, 5103–5114 (2021).
5. Lappalainen, T. et al. Transcriptome and genome sequencing uncovers functional variation in humans. Nature 501, 506–511 (2013).
