# Supplementary material for: Disentangling genetic effects on transcriptional and post-transcriptional gene regulation through integrating exon and intron expression QTLs
Source: Nat Commun. 2024 May 6;15:3786. doi: 10.1038/s41467-024-48244-x (PMC11074126; doi:10.1038/s41467-024-48244-x)
Supplement: Supplementary file 3 — Description of Additional Supplementary Files [file 41467_2024_48244_MOESM3_ESM.pdf]

Description of Additional Supplementary Files

Supplementary Data

File Name: Supplementary Data 1

Description: Top cis-QTL associations with exon levels and intron levels of genes and their ratio detected using QTLtools (Delaneau et al. 2017) and data from 901 LCLs.

File Name: Supplementary Data 2

Description: Genes with shared top cis-QTL signals of different types, and cis-QTL signals of the same type shared between different genes.

File Name: Supplementary Data 3

Description: Cis-QTLs assigned to the first (top) cis-QTL signal by QTLtools (Delaneau et al. 2017) for each cis-QTL type.

File Name: Supplementary Data 4

Description: Best conditional cis-QTLs (top cis-QTL for each rank) identified by QTLtools (Delaneau et al. 2017) for each cis-QTL type.

File Name: Supplementary Data 5

Description: Trans-QTL associations for cis-QTLs of transcription factors (TF), RNA-binding proteins (RBP) and miRNAs.

File Name: Supplementary Data 6

Description: Results from colocalization analysis between different types of top cis-QTLs and variants associated with human traits using the Regulatory Trait Concordance (RTC) method implemented in QTLtools (Delaneau et al. 2017).

File Name: Supplementary Data 7

Description: GTF files with exonic and intronic genomic regions (hg38) covered by only one gene used for exonic and intronic RNA-Seq read counting, respectively, as well as the code to generate these files.
