# Supplementary material for: Disentangling genetic effects on transcriptional and post-transcriptional gene regulation through integrating exon and intron expression QTLs
Source: Nat Commun. 2024 May 6;15:3786. doi: 10.1038/s41467-024-48244-x (PMC11074126; doi:10.1038/s41467-024-48244-x)
Supplement: Supplementary file 11 — Reporting Summary [file 41467_2024_48244_MOESM11_ESM.pdf]

nature portfolio

Corresponding author(s): Anneke Brümmer and Sven Bergmann

Last updated by author(s): Apr 15, 2024

Reporting Summary

Nature Portfolio wishes to improve the reproducibility of the work that we publish. This form provides structure for consistency and transparency in reporting. For further information on Nature Portfolio policies, see our Editorial Policies and the Editorial Policy Checklist.

Statistics

For all statistical analyses, confirm that the following items are present in the figure legend, table legend, main text, or Methods section.

n/a	Confirmed	
☐	☒	The exact sample size (*n*) for each experimental group/condition, given as a discrete number and unit of measurement
☒	☐	A statement on whether measurements were taken from distinct samples or whether the same sample was measured repeatedly
☐	☒	The statistical test(s) used AND whether they are one- or two-sided<br>*Only common tests should be described solely by name; describe more complex techniques in the Methods section.*
☐	☒	A description of all covariates tested
☐	☒	A description of any assumptions or corrections, such as tests of normality and adjustment for multiple comparisons
☐	☒	A full description of the statistical parameters including central tendency (e.g. means) or other basic estimates (e.g. regression coefficient) AND variation (e.g. standard deviation) or associated estimates of uncertainty (e.g. confidence intervals)
☐	☒	For null hypothesis testing, the test statistic (e.g. *F*, *t*, *r*) with confidence intervals, effect sizes, degrees of freedom and *P* value noted<br>*Give P values as exact values whenever suitable.*
☒	☐	For Bayesian analysis, information on the choice of priors and Markov chain Monte Carlo settings
☒	☐	For hierarchical and complex designs, identification of the appropriate level for tests and full reporting of outcomes
☐	☒	Estimates of effect sizes (e.g. Cohen's *d*, Pearson's *r*), indicating how they were calculated

Our web collection on statistics for biologists contains articles on many of the points above.

Software and code

Policy information about availability of computer code

Data collection	RNA-Seq reads were aligned to the human genome (GRCh38) using STAR v2.7 (Dobin et al., Bioinformatics 2013). RNA-Seq reads were counted that mapped uniquely to annotated exons or introns using htseq-count (Anders et al., Bioinformatics 2015). Custom exon and intron annotations, including only genomic regions with no more than one gene annotation on either strand, were generated using bedtools v2.29 (Quinlan and Hall, Bioinformatics 2010). LiftoverVcf from picard-tools (https://broadinstitute.github.io/picard/) was used to lift hg19 genotypes over to hg38. vcf-merge (Danecek et al. Bioinformatics 2011) was used to merge CoLaus and Geuvadis genotypes.
Data analysis	QTLs were mapped using QTLtools v1.3 (Delaneau et al., Nature Communications 2017). Co-localization of QTLs with GWAS variants was analysed using the regulatory trait concordance method (RTC) implemented in QTLtools and using linkage disequilibrium calculated with vcftools (Danecek et al Bioinformatics 2011). coloc (Giambartolomei et al. PLoS Genetics 2014) and q value (Storey 2001) were used to evaluate sharing between cis-QTLs. bedtools v2.29 (Quinlan and Hall, Bioinformatics 2010) was used to determine the overlap between QTLs and between QTLs and genomic regions. Hierarchical clustering of relative effect sizes was performed with scipy.cluster.hierarchy.linkage in Python3 (Virtanen et al. Nature Methods 2020).

For manuscripts utilizing custom algorithms or software that are central to the research but not yet described in published literature, software must be made available to editors and reviewers. We strongly encourage code deposition in a community repository (e.g. GitHub). See the Nature Portfolio guidelines for submitting code & software for further information.

Data

Policy information about availability of data

All manuscripts must include a data availability statement. This statement should provide the following information, where applicable:

- Accession codes, unique identifiers, or web links for publicly available datasets
- A description of any restrictions on data availability
- For clinical datasets or third party data, please ensure that the statement adheres to our policy

The exQTLs, inQTLs and ex-inQTLs (associated with exon and intron expression levels of genes and their ratio, respectively) identified in this study are available as Supplementary Data. Genotypes and RNA-Seq data of the Geuvadis data set are publicly available at ArrayExpress (https://www.ebi.ac.uk/arrayexpress/experiments/E-GEUV-3/). The CoLaus data set is under controlled access and available for researchers through a data transfer agreement (contact: sven.bergmann@unil.ch). Use of data is restricted to pure research and strict compliance with data protection and privacy. Requests from researchers with verifiable credentials (e.g. academic e-mail address) will receive a prompt response (within a week if possible). The fibroblast data set is available through the Gencord data access committee at the European Genome-Phenome Archive (www.ebi.ac.uk/ega; Study ID EGAS00001003485). Gene annotations (genome build GRCh38) were downloaded from GENCODE (version 34, www.gencodegenes.org). Annotations of candidate cis-regulatory elements (cCRE) were downloaded from ENCODE (www.encodeproject.org). Specific molecular QTLs previously identified in LCLs were taken from the supplemental informations of the respective publication for: TF-binding QTLs (Tehranchi et al. Cell 2016), histone modification and DNase hypersensitivity QTLs (Grubert et al. Cell 2015), splicing QTLs (www.gtexportal.org), 3' polyadenylation QTLs (Li et al. Nature Genetics 2021), and protein level QTLs (Battle et al. Science 2015). liftOver chains (from hg19 to hg38) were downloaded from UCSC (http://hgdownload.soe.ucsc.edu). Experimentally determine regulatory sites in LCLs were TF binding clusters (track: TF Clusters downloaded from UCSC Table Browser: https://genome.ucsc.edu/cgi-bin/hgTables), AGO2 binding sites (Wan et al. Nature 2014), PABPC1 and ELAVL1 bound RNAs (downloaded from https://www.ncbi.nlm.nih.gov/geo/; accession codes: GSM944519 and GSM944520). Predicted miRNA target sites were downloaded from TargetScan 43 (www.targetscan.org/vert_80/). miRNA expression levels in LCLs were downloaded from Geuvadis 2 (http://www.ebi.ac.uk/arrayexpress/files/E-GEUV-3/GD452.MirnaQuantCount.1.2N.50FN.samplename.resk10.txt.). A list of TFs was taken from Lambert et al. (Cell 2018) and of RBPs from Sundararaman et al. (Cell 2016). Cis-QTLs for miRNA expression levels were taken from Lappalainen et al. (Nature 2013). 36-mer mappability regions were downloaded from https://www.encodeproject.org/references/ENCSR821KQV/. GWAS associations from different studies were downloaded from the GWAS catalog (https://www.ebi.ac.uk/gwas/; version 1.0).

Research involving human participants, their data, or biological material

Policy information about studies with human participants or human data. See also policy information about sex, gender (identity/presentation), and sexual orientation and race, ethnicity and racism.

Reporting on sex and gender	Sex and gender effects were not analysed in this study.
Reporting on race, ethnicity, or other socially relevant groupings	We restricted our analysis to individuals from Europe or with European ancestry.
Population characteristics	The data of both studies (Geuvadis and CoLaus) was obtained from healthy participants, with similar numbers or males and females.
Recruitment	Information on the recruitment of participants in the Geuvadis and CoLaus studies is available at the original study publications (Lappalainen et al., Nature 2013 and Sönmez Flitman et al., Journal of Proteome Research 2021, respectively).
Ethics oversight	See Geuvadis and CoLaus studies for further informations.

Note that full information on the approval of the study protocol must also be provided in the manuscript.

Field-specific reporting

Please select the one below that is the best fit for your research. If you are not sure, read the appropriate sections before making your selection.

☒ Life sciences ☐ Behavioural & social sciences ☐ Ecological, evolutionary & environmental sciences

For a reference copy of the document with all sections, see nature.com/documents/nr-reporting-summary-flat.pdf

Life sciences study design

All studies must disclose on these points even when the disclosure is negative.

Sample size	No sample size calculations were performed. The sample size was determined by the number of European-descent individuals in the Geuvadis and CoLaus data sets.
Data exclusions	Data from African individuals (participants from Yoruba in Nigeria, labelled YRI) in the Geuvadis data set was excluded, to avoid the results to be potentially dominated by African-Europe-specific genetic effects.
Replication	Replication of different types of cis-QTLs was evaluated by comparing the results obtained independently with the Geuvadis or CoLaus data

Replication	sets. The replication rates were 88-93% for different types of cis-QTLs, when replicating cis-QTLs obtained in the smaller data set (Geuvadis without African samples) in the larger data set (CoLaus).
Randomization	Not relevant for this study. Samples were not allocated into groups.
Blinding	Blinding was not relevant in this study, because samples were not allocated into groups.

Reporting for specific materials, systems and methods

We require information from authors about some types of materials, experimental systems and methods used in many studies. Here, indicate whether each material, system or method listed is relevant to your study. If you are not sure if a list item applies to your research, read the appropriate section before selecting a response.

Materials & experimental systems

n/a	Involved in the study	
☒	☐	Antibodies
☒	☐	Eukaryotic cell lines
☒	☐	Palaeontology and archaeology
☒	☐	Animals and other organisms
☒	☐	Clinical data
☒	☐	Dual use research of concern
☒	☐	Plants

Methods

n/a	Involved in the study	
☒	☐	ChIP-seq
☒	☐	Flow cytometry
☒	☐	MRI-based neuroimaging
